# Administration of Denosumab Preserves Bone Mineral Density at the Knee in Persons With Subacute Spinal Cord Injury: Findings From a Randomized Clinical Trial

**DOI:** 10.1002/jbm4.10375

**Published:** 2020-06-25

**Authors:** Christopher M Cirnigliaro, Michael F La Fountaine, J Scott Parrott, Steven C Kirshblum, Cristin McKenna, Susan J Sauer, Sue A. Shapses, Lihong Hao, Isa A McClure, Joshua C Hobson, Ann M Spungen, William A Bauman

**Affiliations:** ^1^ Department of Veterans Affairs Rehabilitation Research & Development Service National Center for the Medical Consequences of Spinal Cord Injury James J. Peters Veterans Affairs Medical Center Bronx NY USA; ^2^ Department of Physical Therapy, School of Health and Medical Sciences Seton Hall University South Orange NJ USA; ^3^ Departments of Medical Sciences and Neurology Hackensack Meridian School of Medicine at Seton Hall University Nutley NJ USA; ^4^ Department of Interdisciplinary Studies School of Health Professions, Rutgers Biomedical and Health Sciences Newark NJ USA; ^5^ Kessler Institute for Rehabilitation West Orange NJ USA; ^6^ Kessler Foundation West Orange NJ USA; ^7^ Department of Physical Medicine and Rehabilitation Rutgers New Jersey Medical School Newark NJ USA; ^8^ Department of Nutritional Sciences, School of Environmental and Biological Sciences Rutgers University New Brunswick NJ USA; ^9^ Department of Kinesiology and Applied Physiology University of Delaware Newark DE USA; ^10^ Departments of Medicine and Rehabilitation and Human Performance Icahn School of Medicine at Mount Sinai New York NY USA

**Keywords:** BONE MINERAL DENSITY, DENOSUMAB, DUAL ENERGY X‐RAY ABSORPTIOMETRY, IMMOBILIZATION OSTEOPOROSIS, PERIPHERAL QUANTITATIVE COMPUTED TOMOGRAPHY, SPINAL CORD INJURY

## Abstract

Persons with neurologically motor‐complete spinal cord injury (SCI) have a marked loss of bone mineral density (BMD) of the long bones of the lower extremities, predisposing them to fragility fractures, especially at the knee. Denosumab, a commercially available human monoclonal IgG antibody to receptor activator of nuclear factor‐κB ligand (RANKL), may provide an immunopharmacological solution to the rapid progressive deterioration of sublesional bone after SCI. Twenty‐six SCI participants with subacute motor‐complete SCI were randomized to receive either denosumab (60 mg) or placebo at baseline (BL), 6, and 12 months. Areal bone mineral density (aBMD) by dual energy x‐ray absorptiometry (DXA) at 18 months at the distal femur was the primary outcome and aBMD of the proximal tibia and hip were the secondary outcomes analyzed in 18 of the 26 participants (denosumab, *n* = 10 and placebo, *n* = 8). The metrics of peripheral QCT (pQCT) were the exploratory outcomes analyzed in a subsample of the cohort (denosumab, *n* = 7 and placebo *n* = 7). The mean aBMD (±95% CI) for the denosumab versus the placebo groups demonstrated a significant group × time interactions for the following regions of interest at BL and 18 months: distal femoral metaphysis = mean aBMD 1.187; 95% CI, 1.074 to 1.300 and mean aBMD 1.202; 95% CI, 1.074 to 1.329 versus mean aBMD 1.162; 95% CI, 0.962 to 1.362 and mean aBMD 0.961; 95% CI, 0.763 to 1.159, respectively (*p* < 0.001); distal femoral epiphysis = mean aBMD 1.557; 95% CI, 1.437 to 1.675 and mean aBMD 1.570; 95% CI, 1.440 to 1.700 versus mean aBMD 1.565; 95% CI, 1.434 to 1.696 and mean aBMD 1.103; 95% CI, 0.898 to 1.309, respectively (*p* = 0.002); and proximal tibial epiphysis = mean aBMD 1.071; 95% CI, 0.957 to 1.186 and mean aBMD 1.050; 95% CI, 0.932 to 1.168 versus mean aBMD 0.994; 95% CI, 0.879 to 1.109 and mean aBMD 0.760; 95% CI, 0.601 to 0.919, respectively (*p* < 0.001). Analysis of pQCT imaging revealed a continued trend toward significantly greater loss in total volumetric BMD (vBMD) and trabecular vBMD at the 4% distal tibia region, with a significant percent loss for total bone mineral content. Thus, at 18 months after acute SCI, our findings show that denosumab maintained aBMD at the knee region, the site of greatest clinical relevance in the SCI population. © 2020 The Authors. *JBMR Plus* published by Wiley Periodicals LLC. on behalf of American Society for Bone and Mineral Research.

## Introduction

Despite the remarkable clinical strides made in the pharmacological treatment of osteoporosis in several diverse medical conditions, the ability to maintain sublesional bone in individuals with acute motor‐complete forms of spinal cord injury (SCI) has proven to be exceedingly difficult. Over the first couple of years after SCI, 50% to 60% of bone mineral density (BMD) at the epiphyseal and metaphyseal regions of the long bones of the lower extremities may be lost.^(^ ^1^, ^2^ ^)^ Once a large amount of bone has been lost, it would be challenging to restore BMD, trabecular architectural integrity, and mechanical strength to provide sufficient protection against long‐bone fractures of the lower extremities.

The work performed by our group and other investigators to prevent severe bone loss at the knee region shortly after SCI with the administration of i.v. bisphosphonates has not been encouraging.^(^ ^3^, ^4^, ^5^ ^)^ Because of the resulting osteoclastogenesis and unbridled bone resorption that begins within weeks of SCI,^(^ ^6^, ^7^ ^)^ an agent that specifically targets receptor activator of nuclear factor‐κB ligand (RANKL) and its effects to upregulate activity of the osteoclast may prove to be an efficacious approach to maintain skeletal integrity after severe immobilization. Denosumab, a human monoclonal antibody of the IgG_2_ immunoglobulin isotype with a high affinity and specificity for binding RANKL to antagonize its action,^(^ ^8^ ^)^ may afford a novel pharmacological solution to the rapid, progressive deterioration of sublesional skeleton after acute SCI. Gifre and colleagues reported that patients with SCI who were paralyzed for a mean duration of 15 months and received denosumab had suppression in bone turnover and a slight increase in BMD at the hip, which is a noteworthy finding because substantial loss of the sublesional skeleton would have been anticipated.^(^ ^9^ ^)^ Although the report by Gifre and colleagues was provocative, the hip is not the skeletal region of greatest clinical importance because of structural and anatomical considerations in persons with SCI who predominantly fracture at the knee.^(^ ^10^ ^)^ It should also be appreciated that substantial bone mass and structural integrity would have already been lost when treatment was initiated at 15 months after paralysis and immobilization.^(^ ^2^ ^)^

The efficacy of denosumab to preserve skeletal integrity below the level of lesion was tested in a randomized, double‐blinded, placebo‐controlled clinical trial. The primary objective was to measure by dual energy x‐ray absorptiometry (DXA) imaging the areal BMD (aBMD) at the distal femur (distal femoral metaphysis [DFM] and distal femoral epiphysis [DFE]) at 18 months after initiating denosumab administration. The secondary objectives were to measure aBMD at the proximal tibial epiphysis (PTE), femoral neck (FN), and total hip (TH). The exploratory objectives were to determine the metrics of pQCT at the 4% and 38% distal tibia regions 18 months after beginning drug treatment.

## Materials and Methods

### Participants

Participants were recruited from the Kessler Institute for Rehabilitation (KIR, West Orange, NJ, USA) and the James J. Peters Veterans Affairs Medical Center (JJP VAMC, Bronx, NY, USA) within 3 months of acute SCI. All participants completed the study procedures at the study site at which they were enrolled. At the time of enrollment, when the baseline (BL) study drug was administered, all inpatients had motor‐complete SCI (International Standards for Neurological Classification of Spinal Cord Injury [ISNCSCI] grade A and B); one participant converted to motor‐incomplete injury (ISNCSCI grade C) approximately 11 months after acute injury, but remained a wheelchair user without participation in weight‐bearing activities for the remainder of the study. Patients were excluded from participation if they had extensive life‐threatening injuries in addition to SCI, had a history of femur or tibia fracture, had prior bone disease, were men with known hypogonadism prior to SCI, had a history of chronic alcohol abuse, had hyperthyroidism, were pregnant or postmenopausal, had a diagnosis of hypocalcemia or Cushing disease/syndrome, had a current diagnosis of cancer or history of cancer, had heterotopic ossification, had any pre‐existing dental condition or infection, and/or had a known allergy to or prior use of denosumab. The study was performed in agreement with good clinical practice guidelines, and the protocol was approved by the institutional review boards of the KIR and the JJP VAMC. Written informed consent was obtained from each subject prior to study participation. The clinical trial was registered with http://www.ClinicalTrials.gov (Denosumab Administration After Spinal Cord Injury; NCT01983475).

### Clinical trial design

A randomized, double‐blind, placebo controlled, parallel‐group clinical trial was performed from March 2015 to June 2019. A computer‐generated randomization schedule using 13 blocks randomized in a 1:1 manner was generated prior to beginning the study by research pharmacy personnel who were not affiliated with the study protocol in any other manner. Twenty‐six participants were enrolled and randomized to receive either denosumab 60 mg (Prolia; Amgen Inc., Thousand Oaks, CA, USA) or placebo s.c. (identical volume of normal saline s.c.) as soon as possible, but ≤90 days, after acute SCI (BL), with subsequent drug or placebo administration at 6 and 12 months after BL. Enrollment was ended when the planned sample size of 26 was achieved and all possible follow‐up visits were completed. Over the course of the study, all participants and research staff who interacted with participants were blinded to the treatment allocation. To exclude a vitamin D‐deficiency state, levels of 25OHD were measured at BL. In subjects who had vitamin D levels <20 ng/mL at BL, oral supplementation of vitamin D_3_ 4000 IU daily was administered for 30 days with the dose of vitamin D_3_ reduced to 2000 IU/day for the remainder of the study. At BL, each participant had a comprehensive physical examination and laboratory evaluation. The date of the initial study drug infusion was defined as the BL visit. DXA imaging for aBMD was obtained at the BL, 3, 6, 12, and 18 months, and pQCT imaging to measure densitometric, geometry, and strength metrics was performed at BL, 12, and 18 months.

### Outcome measurements

#### Areal *BMD* by *DXA*

The aBMD imaging was obtained by DXA (Lunar iDXA, all software version 16.0; EnCORE, GE Medical Systems, Madison, WI, USA). To minimize interrater variability, a single blinded technician certified by the International Society for Clinical Densitometry (ISCD) performed the acquisition and analysis of the DXA scans. The right and left sides were acquired for each of regions of interest (ROIs) (ie, distal femoral regions, proximal tibia, TH and its subregions); the values for BMD of the sides were averaged, assuming no significant differences were noted. For acquisition of the DFM, DFE, and PTE, commercially available orthopedic knee software supplied by the manufacturer was employed. Analyses of the DFM, DFE, and PTE regions were completed employing a method validated against quantitative computed tomography (QCT) by McPherson and colleagues, with the DXA regions highly correlated to the same regions measured by QCT (*r* ≥ 0.93).^(^ ^11^ ^)^ As a component of the quality assurance procedure, a spine phantom (aluminum spine L1 to L4 encased in acrylic) was scanned more than 300 times over a 5‐year period with the coefficient of variation (CV) <1% (0.54 ± .006%). In accordance with ISCD guidelines, serial DXA scans for precision error were performed and expressed as the least significant change (LSC) of the percent coefficient of variation to assess and quantify changes that may be attributed to random machine error and technician variability.^(^ ^12^, ^13^ ^)^ The LSC was obtained by performing two scans on 30 SCI participants using the “on‐and‐off‐the‐table” method (ie, subjects were repositioned between scans), which yielded the following values for LSC–CV %: FN = 4.0%, TH = 3.0%, DFM = 3.5%, DFE = 4.0%, and PTE = 5.0%.

#### Bone density, geometry, and strength by *pQCT*

The peripheral QCT (pQCT) imaging was performed on the nondominant tibia; measurements were made at the 4% (epiphyseal) and 38% (diaphyseal) regions. A scout view allowed the positioning of cross‐sectional measurements along the tibia moving distal to proximal, with the measurement beginning at the reference line that was placed at the talocrural joint. The pQCT imaging (Stratec XCT 3000; STIM Designs, Carmel, CA, USA) had a slice thickness of 2.4 mm and default voxel size of 0.5 mm, as previously described by Shapses and colleagues.^(^ ^14^, ^15^ ^)^ The CV for pQCT is <2% for volumetric BMD (vBMD) and for the metrics of bone geometry. The following tibial parameters were obtained from the 4% region: total bone mineral content (BMC), total vBMD (mg/cm^3^), trabecular BMC (mg), trabecular vBMD (mg/cm^3^); the following parameters were obtained from the 38% region: total BMC, total vBMD (mg/cm^3^), cortical BMC (mg), cortical vBMD (mg/cm^3^), cortical area (mm^2^), cortical thickness (CoTh; mm), periosteal circumference (PC; mm), endosteal circumference (EC; mm), polar moment of inertia (PMI; mm^4^), and the stress–strain index (SSI; mm^3^). The pQCT outcomes were specified as experimental outcomes prior to the initiation of the study, and as such, they have been included as a Supplementary Table S1.

#### *Laboratory measurements*

Biochemical markers of calcium and vitamin D metabolism were performed at BL. To prevent against alkalization and calcium precipitation, powered boric acid was added to the container for 24‐hour urinary calcium prior to collection; the urinary calcium was measured in the general chemistry laboratory of the VAMC by sequential multiple analyzer (Technicon Instruments, Tarrytown, NY, USA). Commercial laboratories analyzed the serum calcium (LabCorp, Raritan, NJ, USA) and 25OHD levels (immunoassay; Quest Diagnostics, Teterboro, NJ, USA). The sensitivity of the 25OHD assay was 4 ng/mL; the within‐assay coefficients of variation were not available from Quest Diagnostics.

### Statistical analyses

From prior work without pharmacological intervention at 18 months after acute SCI, the loss of BMD at the distal femur is 16.3 ± 0.3%. To date, no reports have reported the effect of initiating treatment with denosumab on sublesional bone in the subacute period after SCI. Of note, no bone‐sparing effect at the knee (distal femur and proximal tibia) was observed with the administration of pamidronate or zoledronic acid.^(^ ^3^, ^4^ ^)^ If a 30% reduction at the distal femoral region was postulated to be observed with denosumab administration, the study is well‐powered with a count of 12 (94%; *Z*‐power = 3.99) or with 9 (85.6%; *Z*‐power = 2.00).^(^ ^16^ ^)^ The secondary outcomes included aBMD measurements for the other ROIs of the leg. The exploratory outcomes were the parameters of skeletal architecture and geometry at the distal tibia by pQCT with the stated objective to collect new information and empirical data to plan for future investigations. This study was not powered for the secondary and exploratory outcomes.

Values are expressed as group mean and 95% CI or SD, where applicable. The primary outcome measure was aBMD at the distal femur (DFM and DFE) at 18 months after initiating denosumab administration. The secondary outcome measurements were aBMD at the PTE, FN, and TH, and the exploratory outcomes were the pQCT metrics at the 4% and 38% distal tibia regions at 18 months after beginning drug treatment. Independent *t* tests were performed on BL values to identify group (denosumab versus placebo) differences for demographic and clinical characteristics; DXA measurements; and pQCT densitometric, geometry, and strength values. The mean difference in aBMD between right and left sides at all skeletal regions of interest (ROI) (eg, DFM, DFE, PTE, FN, and TH) was compared with the null hypothesis (zero) using a one‐sample analysis. Because no significant differences were found between the left and right side, the two sides were averaged and presented as a single value for each region. To identify changes across time, DXA values were compared using a 5 × 2 (time × treatment group) repeated measures analysis of covariance (RANCOVA) using race as a covariate. The exploratory values for pQCT were compared using a 3 × 2 RANCOVA using baseline geometry values and race as a covariate. Missing values were imputed to optimize the analysis of the available data; linear regression was used to impute the month‐12 value for two participants in the denosumab group, and for one participant in the control group. To determine if assumptions of sphericity were met within the levels of our independent variable, Mauchly's test was performed, and if determined to be statistically significant (*p* < 0.05), the Greenhouse‐Geisser correction was applied to determine the significance of all main effects. To further characterize significant within‐group differences from BL at various time points, post hoc pairwise comparisons were performed. To control for the type‐I error rate, a Bonferroni correction was applied, and a priori level of significance was set at *p* ≤ 0.0125 for DXA and *p* ≤ 0.025 for the multiple comparisons from the post‐hoc analyses by RANCOVA for pQCT; for all additional analyses, the level of significance was set at *p* ≤ 0.05. Percent change variables were created in each group at 3, 6, 12, and 18 months for the DXA outcomes and compared between the treatment and placebo groups by independent *t* tests. Statistical analyses were completed using IBM SPSS Statistics version 22 (IBM, Armonk, NY, USA); graphs were generated by Prism GraphPad (version 8.1 for Windows; GraphPad Software, San Diego, CA, USA).

## Results

### Participant characteristics

**Figure 1:**
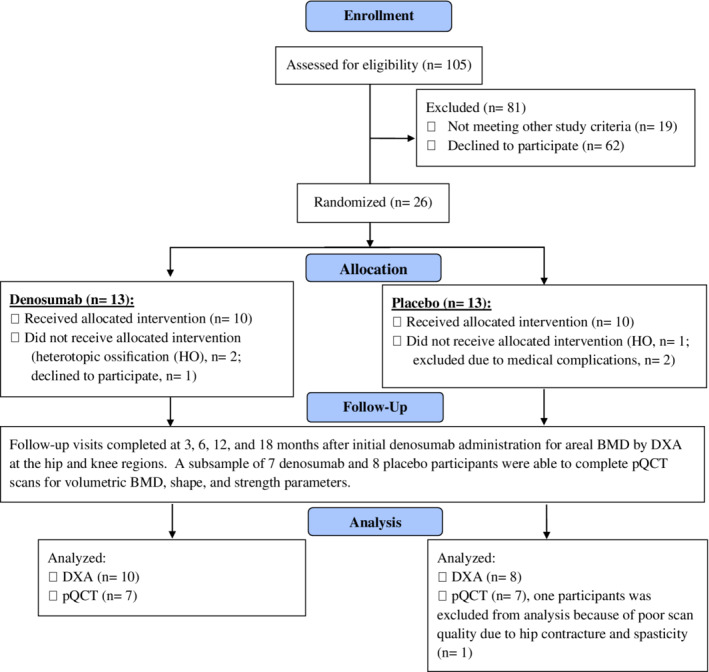
CONSORT (Consolidated Standards of Reporting Trials) diagram of participant flow.

Twenty‐six participants were enrolled and randomized into the study, with 13 participants randomized to either the active drug or the placebo group for BL measurements. Ten participants in the denosumab group and eight participants in the placebo group completed the follow‐up visits at 3, 6, 12, and 18 months. Because of the logistical barrier that required participants to travel to a distant facility to perform the pQCT measurements, a subsample of seven denosumab and seven placebo participants completed pQCT imaging at the 12‐ and 18‐month visits (Fig. 1). The denosumab and placebo groups were well‐matched for most BL demographic characteristics, with no significant group differences found for any categorical or continuous variables (Table 1). In the treatment group, the time after acute SCI to denosumab administration was 70 ± 19 days, and in the placebo group, 76 ± 18 days. One of the 10 participants in the denosumab group and 3 of the 8 participants in the placebo group participated in inpatient and/or outpatient rehabilitation that included functional electrical stimulation cycling for 1 to 2 day/week. At BL, calcium and vitamin D metabolism was not significantly different between the denosumab and placebo groups (Table 1).

**Table 1:** Demographics and Clinical Characteristics of the Study Participants

	Denosumab (*n* = 10)	Placebo (*n* = 8)
Age (years)	33.7 ± 11.3	33.9 ± 12.3
Height (m)	1.76 ± 0.1	1.74 ± 0.1
Weight (kg)	92.0 ± 31.6	84.0 ± 15.5
BMI (kg/m^2^)	29.5 ± 9.5	27.8 ± 5.4
Sex (men: women), *n*	8/2	8/0
Race (white: black: Latino), *n*	5/4/1	4/0/4
Paraplegia/tetraplegia, *n*	6/4	6/2
ISNCSCI (A/B/C), *n*	7/2/1	7/1/0
Days from acute SCI to BL	70.4 ± 18.9	76.1 ± 17.7
FES Cycling, *n* (%)	1 (10)	3 (37.5)
Smoker, *n* (%)	3 (30)	2 (25)
Alcohol use, *n* (%)	5 (50)	2 (25)
Corticosteroids after SCI, *n* (%)	0 (0)	1 (12.5)
Opioid use, *n* (%)	4 (40)	6 (75)
25OHD (ng/mL)	30.3 ± 14.1	23.0 ± 10.5
iPTH (pg/mL)	12.3 ± 5.3	12.4 ± 5.1
Urine calcium (mg/24 hours)	246.5 ± 182.1	212.4 ± 113.5
Serum calcium (mg/dL)	9.4 ± 0.4	9.6 ± 0.6

Values are presented as group mean ± SD. There were no significant differences between groups for any of the characteristics presented.

BL = Baseline; DOI = duration of injury; FES = functional electrical stimulation; iPTH = intact parathyroid hormone; ISNCSCI = International Standards for Neurological Classification of Spinal Cord Injury; OH = hydroxy; SCI = spinal cord injury.

### DXA outcome measurements

The groups were well‐matched for BL aBMD at the hip and knee, with no significant differences noted between the groups (Table 2). The aBMD values at the DFE revealed a trend toward a significant main effect for group, which was not present at any of the other aBMD regions. A significant main effect for time and treatment group–time interaction was observed for the ROIs (ROIs; DFE, DFM, PTE, FN, and TH), suggesting a sparing of aBMD over time in the denosumab group with a significant loss of aBMD in the placebo group. Within‐group paired analysis revealed a significant decrease from BL in the placebo group that started at month 3 for the DFE, DFM, and TH, at month 6 for the FN, and at month 12 for the PTE, with continued progressive and significant demineralization at 12 and 18 months for all the skeletal ROIs (Table 2).

**Table 2:** Areal BMD Measurements at Regions of Interest in Participants With Subacute Spinal Cord Injury Administered Denosumab or Placebo

DXA variables	Treatment groups	Baseline	Month 3	Month 6	Month 12	Month 18	Main effect (*p* value)		
							Time	Interaction	power
aBMD (g/cm^2^)									
DFM	Denosumab	1.187 (1.074–1.300)	1.201 (1.079–1.323)	1.191 (1.074–1.308)	1.180 (1.066–1.294)	1.202 (1.074–1.329)	0.001	0.001	0.94
	Placebo	1.162 (0.962–1.362)	1.113 (0.927–1.300)	1.058 (0.887–1.229)^*^	1.008 (0.823–1.193)^**^	0.961 (0.763–1.159)^**^			
DFE	Denosumab	1.557 (1.437–1.675)	1.571 (1.453–1.689)	1.540 (1.412–1.668)	1.541 (1.406–1.677)	1.570 (1.440–1.700)	<0.001	<0.001	1.0
	Placebo	1.565 (1.434–1.696)	1.476 (1.300–1.652)^*^	1.365 (1.162–1.568)^**^	1.185 (0.982–1.388)^***^	1.103 (0.898–1.309)^***^			
PTE	Denosumab	1.071 (0.957–1.186)	1.074 (0.964–1.184)	1.058 (0.942–1.174)	1.052 (0.933–1.172)	1.050 (0.932–1.168)	<0.001	0.001	0.99
	Placebo	0.994 (0.879–1.109)	0.959 (0.820–1.098)	0.920 (0.778–1.063)	0.845 (0.676–1.014)^*^	0.760 (0.601–0.919)^**^			
FN	Denosumab	1.112 (0.977–1.247)	1.127 (0.993–1.261)	1.102 (0.959–1.245)	1.159 (1.009–1.309)	1.144 (0.993–1.295)	<0.001	<0.001	1.0
	Placebo	1.094 (0.951–1.237)	1.034 (0.886–1.189)^**^	0.982 (0.840–1.132)^**^	0.912 (0.776–1.137)^***^	0.875 (0.705–1.045)^***^			
TH	Denosumab	1.110 (0.978–1.241)	1.130 (0.992–1.267)	1.136 (0.948–1.324)	1.151 (0.966–1.335)	1.151 (0.969–1.333)	<0.001	<0.001	1.0
	Placebo	1.115 (1.012–1.218)	1.023 (0.940–1.128)^***^	0.957 (0.873–1.078)^***^	0.875 (0.775–1.054)^***^	0.833 (0.707–0.959)^***^			

Values are expressed as group mean ± 95% CI. *p* Value represents significant omnibus effect from RANCOVA. There was no group main effect for any of the DXA variables presented. Significant decrease from baseline for post hoc paired comparisons.

aBMD = areal bone mineral density; DFE = distal femoral epiphyses; DFM = distal femoral metaphysis; FN = femoral neck; PTE = proximal tibial epiphysis; TH = total hip.

^*^ *p* ≤ 0.0125.

^**^ *p* ≤ 0.01.

^***^ *p* ≤ 0.001.

**Figure 2:**
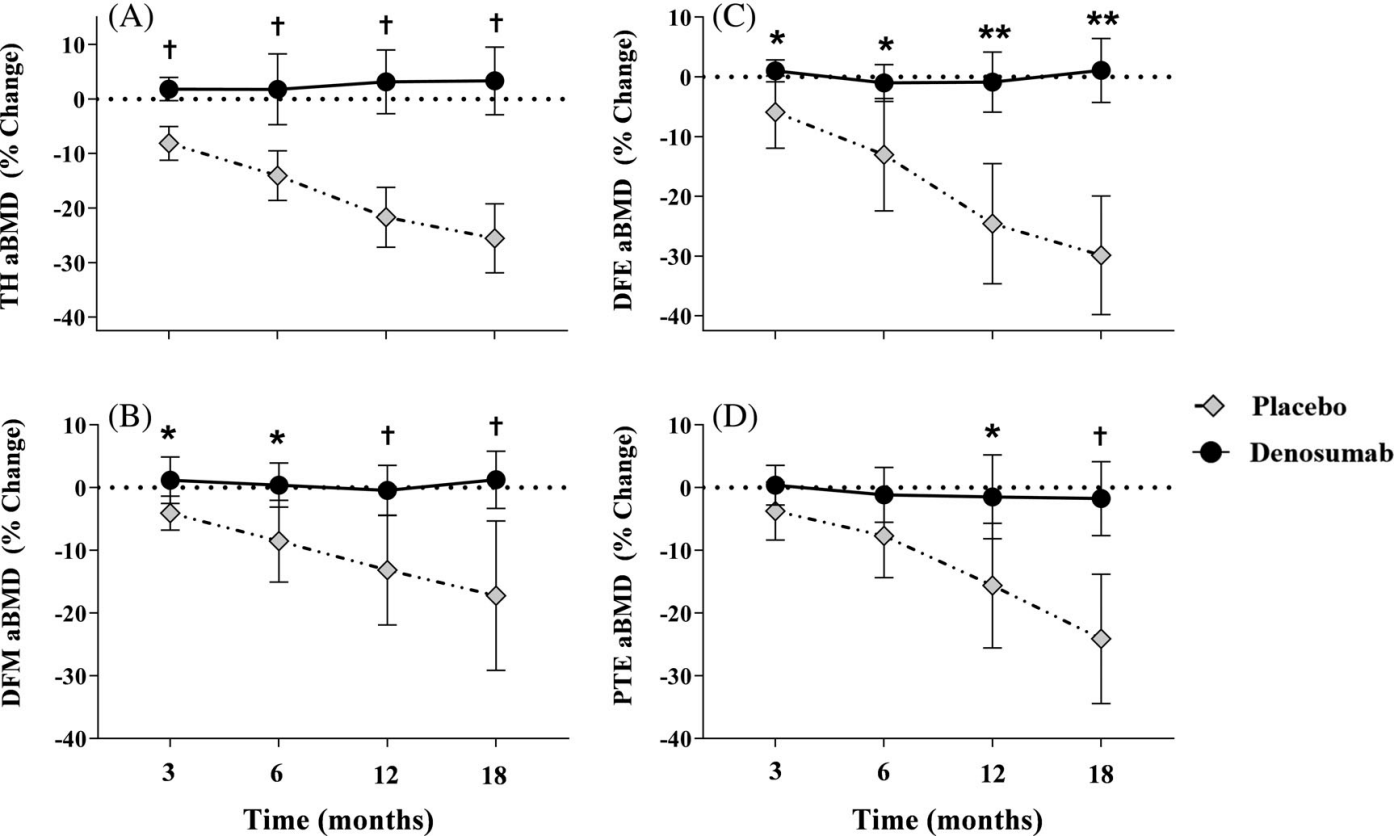
Percent change from baseline in areal BMD at the (*A*) total hip (TH), (*B*) distal femur metaphysis (DFM), (*C*) distal femur epiphyses (DFE), and (*D*) proximal tibia epiphysis (PTE). Percent change from baseline for denosumab versus placebo at the respective time points: ^*^ *p* < 0.05; ^†^ *p* < 0.01; ^**^ *p* < 0.001.

**Figure 3:**
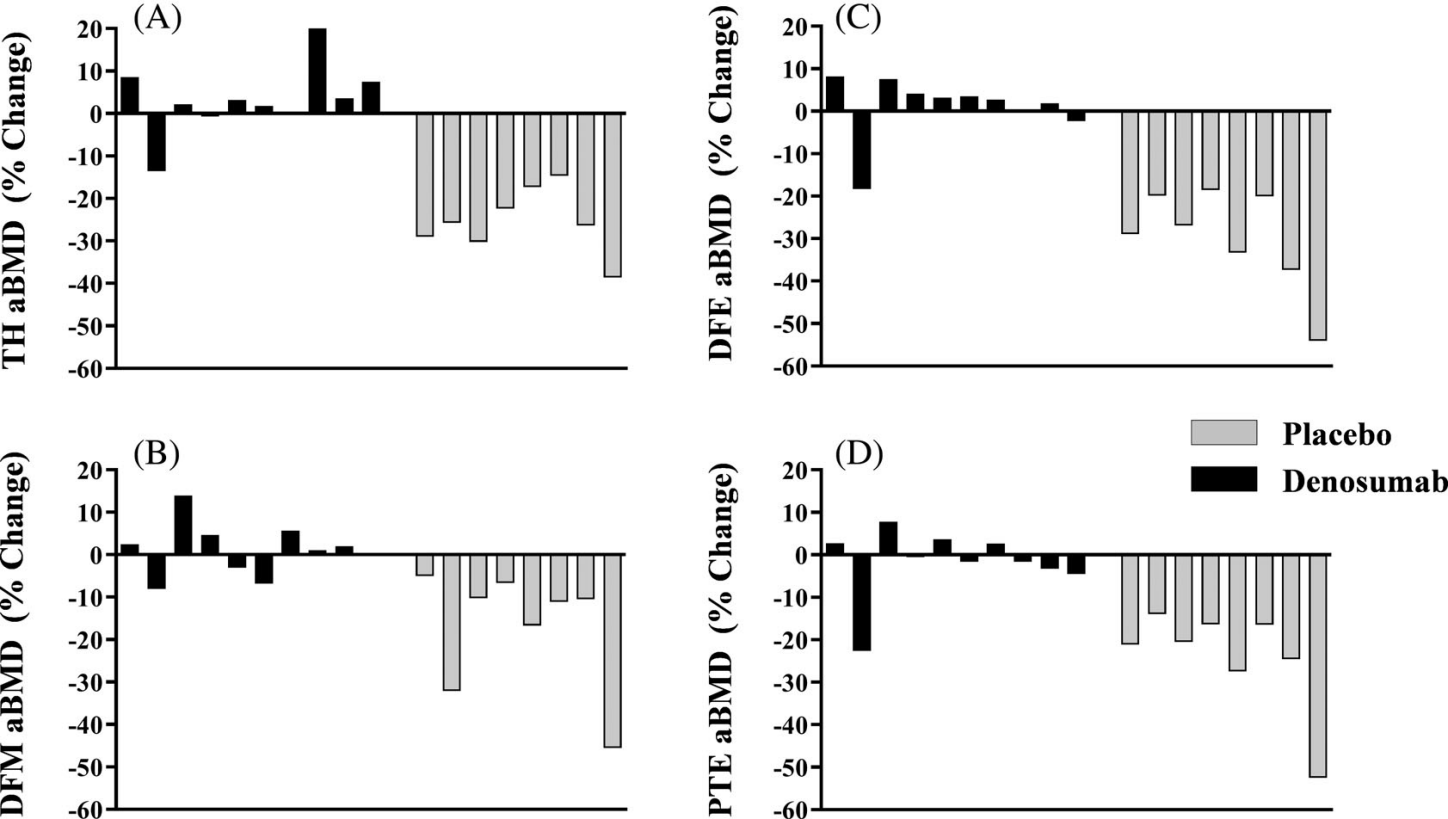
Individual participant percent change from baseline in areal BMD at the (*A*) total hip (TH), (*B*) distal femur metaphysis (DFM), (*C*) distal femur epiphyses (DFE), and (*D*) proximal tibia epiphysis (PTE).

Compared with the denosumab group, the percent change from baseline to months 3 and 6 revealed that the placebo group lost a significantly greater percentage of aBMD at the TH, DFM (Fig. 2A,B), DFE, and PTE (Fig. 2C,D). At months 12 and 18, compared with the denosumab group, percent aBMD declined further in the placebo group at the TH (3.1% ± 8.2 versus −21.7% ± 6.6, and 3.3% ± 8.7 versus −25.6% ± 7.6, respectively; *p* < 0.001 and *p* < 0.001), DFM (−0.4% ± 5.6 versus −13.2% ± 10.4, and 1.2% ± 6.4 versus −17.2% ± 14.2, respectively; *p* = 0.004 and *p* = 0.002) (Fig. 2*A*,*B*), DFE (−0.9% ± 7.0 versus −24.6 ± 12.0, and 1.1% ± 7.5 versus −30.0% ± 11.9, respectively; *p* < 0.001 and *p* < 0.001), and PTE (−1.5% ± 9.4 versus −15.6% ± 11.9, and −1.7% ± 8.2 versus −24.1% ± 12.3, respectively; *p* = 0.012 and *p* < 0.001) (Fig. 2*C*,*D*). Individual percent change from BL values are presented at 12 and 18 months at the TH, DFM, DFE, and PT for participants in the denosumab and placebo groups (Fig. 3A–D). At month 18 in the denosumab group, one participant lost between 8% to 23% of BL aBMD at the hip and knee ROI and five participants lost <7% of BL aBMD at the hip and knee regions. At 18 months, all of the control‐group participants lost >10% of BL aBMD at the hip (six lost >20%), DFE (six lost >20%), and PTE (five lost >20%); the DFM lost the least amount of aBMD, but six participants still lost >10% of BL aBMD at 18 months (range: −10.2% to −45.6%) (Fig. 3). Thus, aBMD at knee and hip regions was generally maintained in the denosumab group, whereas aBMD was progressively lost in the placebo group.

### pQCT outcome measurements

A subset of 14 participants from the 18 participants who completed the DXA outcomes also successfully completed the pQCT imaging measurements. At BL, the groups were well‐matched for all pQCT densitometric, geometry, and strength variables at the 4% and 38% tibial regions with no significant differences noted between the groups. As a result of the reduced number of participants who were able to complete the pQCT imaging, which resulted in the loss of statistical power when performing the analysis, the pQCT analysis revealed nominal significance for all skeletal metrics assessed. At the 4% distal tibia, a significant main effect for group, time, and group–time interaction was observed for total BMC, with a significant main effect for time observed for vBMD. Analysis of trabecular bone at the 4% region revealed a significant main effect for time for BMC and vBMD, with a trend toward a significant group–time interaction effect for vBMD. Within‐group paired analysis at the 4% region revealed a significant main effect for group for vBMD and a significant decrease from BL in the placebo group for total BMC at month 18 (*p* = 0.009). At the 38% tibia shaft region, a significant main effect for group and time was observed for total BMC. Analysis of the cortical bone compartment at the 38% region revealed a significant main effect for group and time for cortical BMC and vBMD. Analysis of bone geometry metrics (cortical area, CoTh, PC, and EC) provided values that were not significantly different between the denosumab and placebo groups at months 12 and 18. Despite the geometry metrics in both groups not being significantly different, a significant main effect for group and time was observed for the strength metrics (PMI and SSI; Supplementary Table S1).

### Adverse events

Over the course of the study, only one adverse event, worsening of psoriasis, was related to the study drug; no serious drug‐related adverse events were observed adverse events were observed. Furthermore, no infection‐related adverse events (ie, urinary, respiratory) were observed over the course of the study.

## Discussion

A randomized placebo‐controlled clinical trial was performed in persons with a subacute motor‐complete SCI to determine the efficacy of denosumab to preserve aBMD at the knee. Of note, denosumab generally maintained aBMD at the distal femur and proximal tibia in persons with a subacute motor‐complete SCI. These findings were supported by the trend toward preservation of total and trabecular vBMD at the 4% tibial region by pQCT. From this trend in pQCT‐derived trabecular and cortical vBMD measurements over the course of the study, the robust aBMD differences from the primary analysis are most likely caused by the mitigation of bone loss within the the trabecular compartment. Although long‐bone fractures of the lower extremities were not a study endpoint, nor could they be determined after acute SCI in this relatively short clinical trial, our findings would be anticipated to represent a major advance in the prophylactic prevention of sublesional bone loss and subsequent fractures in persons with chronic SCI.

Bone loss associated with severe, prolonged immobilization has been exceedingly difficult to prevent. In 1988, the National Institute on Disability and Rehabilitation Research reported that 3.8% of the US population was estimated not to be able to perform any major activity.^(^ ^17^ ^)^ At the lowest end of the activity and weight‐bearing spectrum, SCI results in a marked and rapid bone deterioration below the level of lesion that is far greater than that which occurs in other conditions associated with bone loss, such as postmenopausal osteoporosis, bedrest, and space flight.^(^ ^1^, ^2^, ^18^, ^19^, ^20^ ^)^ Appendicular fractures are a frequent complication in individuals with SCI and commonly occur at the distal femur and proximal tibia, which corresponds to the skeletal regions with substantial bone loss and at high risk of sustaining trauma because of anatomical considerations. Falls from a wheelchair and transfers are the most common causes of fracture,^(^ ^10^ ^)^ but fractures may occur when performing routine activities of daily living as well. From a chart review performed that captured 7590 male veterans with chronic traumatic SCI, 155 fractures were identified in 140 patients with the majority of fractures occurring of the femur (*n* = 52; 33%) and tibia/fibula (*n* = 83; 54%); 67 fractures (43%) occurred while in a wheelchair, of which 41 (22%) occurred during transfers to or from the wheelchair.^(^ ^21^ ^)^ The incidence of lower extremity fractures in those with SCI rises with duration of paralysis, with an incidence of 1.0% during the initial year after injury and rising to 4.6% after 20 years of injury.^(^ ^22^, ^23^ ^)^ A few investigators have attempted to identify cutoff points for the risk of fracture in the SCI population. In a study of 41 persons with SCI of whom 14 had sustained fractures, the risk of fracture at the FN increased 2.2 and 2.8 times for each 0.1 gm/cm^2^ decrement in BMD or for each unit decrease in the SD (*T* value), respectively.^(^ ^24^ ^)^ A correlation between epiphyseal trabecular BMD has been inversely related to the risk of fracture.^(^ ^23^ ^)^

Two broad classifications of agents are currently approved by the US Food and Drug Administration (FDA) for the treatment of osteoporosis: antiresorptive agents and anabolic agents. The antiresorptive agents include bisphosphonates and a human monoclonal antibody to RANKL (denosumab). The anabolic agents are parathyroid hormone 1–34 (teriparatide), an analog of parathyroid hormone‐related peptide (abaloparatide), and a human monoclonal anti‐sclerostin antibody (romosozumab).

The efficacy of bisphosphonates has been tested in various preclinical models of immobilization.^(^ ^25^ ^)^ Bisphosphonate administration was not effective at preventing bone loss or maintaining cortical bone strength in the tail‐suspension model in the rat.^(^ ^26^ ^)^ Treatment with a bisphosphonate had only a modest effect at preserving cortical bone mass and mechanical properties during long‐term disuse on metacarpal diaphyses in dogs,^(^ ^27^ ^)^ which is in contrast with the more dramatic action of the bisphosphonate administration in other conditions associated with osteoporosis. Thus, these findings from preclinical models of immobilization that tested bisphosphonate administration serve to differentiate the efficacy of treatment of disuse osteoporosis from other models of osteoporosis. In SCI rats, upregulation of RANKL expression may be largely responsible for the increase in bone resorption,^(^ ^28^ ^)^ which may be the reason that antagonism of RANKL with denosumab in the work herein was found to be more effective than treatment with bisphosphonates in our prior reports.^(^ ^3^, ^4^ ^)^

Bisphosphonates have proven to be less efficacious in several clinical models of immobilization.^(^ ^3^, ^4^, ^25^, ^29^ ^)^ Two case series with a fairly small number of patients suggested that there is a benefit of bisphosphonate administration to preserve bone in persons with motor‐incomplete SCI who can bear weight and ambulate, if only to a limited degree.^(^ ^30^ ^)^ In another study of subjects with SCI, most of whom regained the ability to ambulate, alendronate preserved bone at the femoral shaft and total leg to at least 18 months after initial paralysis.^(^ ^31^ ^)^ Much of the literature that reported the effect of bisphosphonates on BMD after acute SCI has been performed in cohorts comprised of a mixture of patients with varying degrees of neurological impairment (eg, motor‐complete and motor‐incomplete injuries) and hence, the ability to ambulate. However, our experience, as well as work by others, has raised questions of the efficacy of bisphosphonate administration in persons with neurologically more motor‐complete forms of SCI—that is, in those who lack the ability to walk.^(^ ^3^, ^4^, ^5^, ^32^ ^)^ Investigators have reported varying degrees of success with bisphosphonate administration at the hip in patients after motor‐complete forms of acute SCI, but generally studies have not addressed the efficacy of bisphosphonates, or have found them not to be effective, at the knee in persons with motor‐complete lesions.^(^ ^4^, ^32^ ^)^ Because the knee is the region that is at heightened risk of fracture in those with SCI,^(^ ^10^, ^21^ ^)^ the use of bisphosphonates to preserve bone at the hip may be considered of lesser clinical relevance.

The effects of denosumab on bone remodeling are suggested to be more potent than those of bisphosphonates by histomorphometric, bone turnover, and bone density measures.^(^ ^33^, ^34^, ^35^ ^)^ In *in vivo* and *in vitro* studies, unloading of the hindlimbs of rats and in a rodent models of spinal cord transection have been shown to increase RANKL expression and translation.^(^ ^36^, ^37^, ^38^ ^)^ Of some interest, in a mouse model of hindlimb suspension that had an osteocyte conditional deletion of RANKL, bone loss was prevented.^(^ ^39^ ^)^ Thus, osteocytes are the predominant cellular source of RANKL for osteoclast differentiation, activity, and viability; probably, in large part, they are responsible for the rapid bone resorption that occurs in patients after SCI. Therefore, antagonism of RANKL action may be anticipated to play a major role in the prevention of bone deterioration in states of severe immobilization. Because of the marked osteoclastosis and bone resorption that develops in patients shortly after SCI,^(^ ^40^ ^)^ the dramatic reduction in eroded bone surfaces with treatment with denosumab observed in the treatment of postmenopausal osteoporosis may be relevant to its effect to prevent or reduce bone loss in patients with more severe forms of immobilization. In a prospective study of 23 male patients with a duration of SCI of 99 ± 30 days at baseline who were administered denosumab, higher serum levels of RANKL correlated inversely with TH BMD at 6 months; after beginning administration of denosumab, RANKL levels were undetectable in two‐thirds of the participants and, when bone should have been progressively lost, drug treatment essentially maintained BMD at the TH and FN.^(^ ^41^ ^)^ In another report by the same group of investigators, 14 patients with SCI were treated for 12 months with denosumab that was begun, on average, 15 months after acute SCI; a slight increase in BMD at the hip and an associated suppression in biomarkers of bone resorption and formation were observed.^(^ ^9^ ^)^ Neither of these articles by Grife et al. reported imaging findings at the knee after denosumab administration.^(^ ^9^, ^41^ ^)^

The ability of teriparatide to preserve bone below the level of lesion at time of acute SCI has not been rigorously addressed, although some work was reported in those with chronic SCI after teriparatide and/or mechanical interventions (robotically assisted gait training or vibration) with the authors concluding that the therapeutic effect was not of clinical significance for those with SCI.^(^ ^42^, ^43^ ^)^ When used to treat postmenopausal osteoporosis, abaloparatide appears to be more effective to increase BMD than teriparatide, with the difference in action between these two drugs especially evident for the appendicular skeleton where abaloparatide appeared to reduce nonvertebral fractures, a benefit that has not been observed with teriparatide therapy;^(^ ^44^, ^45^, ^46^ ^)^ as such, there is the possibility that abaloparatide may be beneficial to preserve sublesional bone in persons after acute SCI or restore sublesional bone in those with more long‐standing SCI.

In April 2019, the FDA approved romosozumab for the treatment of postmenopausal osteoporosis. Preclinical studies of sclerostin antagonism or genetic ablation in acute and chronic rodent models of SCI have been performed by our group.^(^ ^47^, ^48^, ^49^ ^)^ In a rat model, anti‐sclerostin antibody (reagent provided by Amgen) was begun 7 days after spinal cord transection (significant bone loss occurs as early as 7 days after SCI in the rat model) and the agent was then administered weekly over the next 7 weeks; sclerostin antagonism completely prevented and/or reversed the marked bone loss that occurred in the untreated spinal cord transected animals.^(^ ^47^ ^)^ Sclerostin knockout mice (animals were provided by Amgen) that underwent spinal cord transection were protected from the severe sublesional bone loss that occurred in WT mice with spinal cord transection.^(^ ^48^ ^)^ To test whether sclerostin antagonism could reverse bone loss in rats after chronic motor‐complete SCI, treatment was initiated with anti‐sclerostin antibody or vehicle 12 weeks after spinal cord transection and continued for 8 weeks.^(^ ^49^ ^)^ In SCI rodents that received normal saline injections, there was significant reduction in BMD, estimated bone strength, and deterioration of bone structure at the distal femoral metaphysis at 20 weeks, whereas animals that received anti‐sclerostin antibody had remarkably restored BMD, bone structure, and bone mechanical strength.^(^ ^49^ ^)^ Because the Wnt signaling system is inhibited in preclinical models of SCI because of increases in two inhibitors of this signaling pathway, sclerostin and Dickkopf‐related protein 1 (DKK1),^(^ ^50^ ^)^ it may be speculated that treatment restoring this vital skeletal pathway to a more normal level of activity would be more favorable to bone health than merely suppressing osteoclast function. In further support of this argument, osteocyte number and shape, cellular orientation, number and length of dendritic projections, and, possibly, viability was improved with sclerostin antagonism.^(^ ^47^ ^)^ Of note, osteocyte apoptosis was found to be essential to activate bone remodeling by triggering osteocyte RANKL expression in response to hindlimb unloading.^(^ ^36^ ^)^ As such, the long‐term health of the sublesional skeleton may benefit from this, as yet untested, therapeutic approach of sclerostin antagonism after acute paralysis.

Currently, clinicians have not been provided with any consistent guidance as to what agent, if any, should be considered in the prevention of bone loss in persons with recent SCI. Despite an acknowledgment in the literature that support is lacking,^(^ ^51^ ^)^ many physicians continue to prescribe bisphosphonates for patients with acute/subacute and chronic SCI, presumably because this class of drugs is widely used as a first‐line treatment in other forms of osteoporosis and that bisphosphonates have been reported to maintain or increase bone at the hip in those with recent SCI.^(^ ^5^, ^31^, ^32^ ^)^ As evidence against this clinical approach in persons with more neurologically severe forms of motor lesion, the work by our group has shown a lack of efficacy of zoledronic acid to prevent bone loss at the knee, and its administration may, paradoxically, be associated with increased bone loss at the knee while preserving bone at the hip, which is not the desired outcome in the treatment of an SCI population. The findings from the work presented herein strongly suggest that treatment with denosumab initiated within several months of acute SCI may be an efficacious approach to maintain BMD at the knee. Importantly, discontinuing denosumab has been amply demonstrated to result in rapid bone loss.^(^ ^52^, ^53^, ^54^, ^55^ ^)^

Because treatment with denosumab has been demonstrated to be well‐tolerated for extended periods of time in postmenopausal women^(^ ^56^ ^)^ and skeletal histomorphometry remains normal throughout this period,^(^ ^57^ ^)^ there may be a strong clinical incentive to treat patients with SCI for an open ended period with this agent to preserve bone integrity until another agent that proves to be more efficacious for bone health in the SCI population is identified, albeit there are risks associated with long‐term exposure to antiresorptive medications.^(^ ^58^, ^59^, ^60^, ^61^ ^)^

There were several limitations to the work presented herein. Although the sample size was relatively limited, it was more than sufficient to detect a significant change for the primary endpoint of aBMD at the distal femur after denosumab administration in persons with subacute SCI, as well as of ample size to determine the significance of several other secondary endpoints obtained by DXA imaging for the ROI of the lower extremity. Considering the observed trends, the inability to find significant differences between the denosumab and placebo groups for the pQCT measurements was most likely because of the lack of sufficient statistical power caused by an insufficient size of the subgroups captured for imaging. The size of the sample did not allow the determination of the effect of denosumab therapy for the demographic variables of age, gender, or race. A comparison of incident fractures of the lower extremities between the groups, with a reduction in fractures that would have been anticipated to have occurred in the active drug group compared with the placebo group, was not possible because of the limited duration of the study, as well as the small sample size. Finally, a study design that allowed for periodic assessment of biomarkers of bone turnover at the appropriate time intervals may have provided additional insight into the mechanisms responsible for changes in the metrics of bone imaging over the duration of the study. Several of the shortcomings of this work could be addressed in a larger, randomized clinical trial designed to have a broad diversity of ages and races composed of both men and women followed for a longer length of time.

Bone resorption is greatest at the knee region (eg, epiphyseal and metaphyseal regions of the femur and tibia) in the first couple of years after injury.^(^ ^1^, ^62^, ^63^ ^)^ As demonstrated in the study herein, denosumab successfully prevented bone loss at the distal femur and proximal tibia in the majority of participants with motor‐complete SCI. However, in the participants who lost bone at the knee, it may be speculated that shorter intervals between drug dosing or higher doses of denosumab at 6‐month intervals may have prevented the observed greater degree of bone loss in the few more drug‐resistant participants. Although not routinely recommended in the treatment of other forms of osteoporosis, obtaining serial bone biomarkers of resorption may serve to identify those individuals with subacute SCI who may have benefited from the administration of higher or more frequent dosage of denosumab.

The pharmacological therapeutic approach tested in this study is the first to prevent bone loss at the knee in persons with subacute motor‐complete SCI. As such, the administration of denosumab provides a practical strategy for the clinician to maintain bone mass in persons with recent SCI. Improvements in sublesional bone BMD would be anticipated to improve confidence to engage in personal activities (eg, recreational endeavors and independence). The quality of life would be substantially improved because of the ability to engage more confidently in activities of daily living and to more fully integrate into the community. The ability to improve sublesional bone health will help to ensure that persons will qualify to participate in upright walking‐assisted activities, such as use of powered exoskeletal robots or epidural stimulation for ambulation. To avoid fragility fracture, improved bone health will also be a necessary clinical component of any future neuro‐regenerative strategies that restore the ability to ambulate in persons with SCI.

## Disclosures

All authors have no conflict of interest to report.

### Peer Review

The peer review history for this article is available at https://publons.com/publon/10.1002/jbm4.10375.

## Supporting information


**Appendix S1**: Supporting information
