# Supplementary material for: Administration of Denosumab Preserves Bone Mineral Density at the Knee in Persons With Subacute Spinal Cord Injury: Findings From a Randomized Clinical Trial
Source: JBMR Plus. 2020 Jun 25;4(8):e10375. doi: 10.1002/jbm4.10375 (PMC7587457; doi:10.1002/jbm4.10375)
Supplement: Supplementary file 1 — Appendix S1: Supporting information [file JBM4-4-e10375-s001.docx]

| **Supplementary Appendix 1: Table 1. Total, Trabecular, and Cortical Volumetric Bone Mineral Density, Bone Geometry and Strength Values of the Tibia in Participants with Subacute Spinal Cord Injury Administered Denosumab or Placebo** | | | | | | | |
| --- | --- | --- | --- | --- | --- | --- | --- |
| **pQCT**  **Variables** | **Treatment**  **Groups** | **Baseline** | **Month 12** | **Month 18** | **Group** | **Main Effect**  **(p-value)** | **Interaction** |
|  |  |  |  |  |  | **Time** |  |
| **Total (4% site)**  vBMD (mg/cm^3^) | Denosumab | 336.1 (294.1 - 378.1) | 330.1 (274.1 - 386.1) | 334.6 (277.9 - 391.2) | NS | 0.03 | 0.055 |
|  | Placebo | 330.3 (284.4 - 376.2) | 279.1 (210.8 - 347.5)^a^ | 274.2 (202.4 - 346.0)^b^ |  |  |  |
| BMC (mg) | Denosumab | 422.2 (371.7 - 472.8) | 398.6 (334.0 – 463.2) | 424.2 (367.9 – 480.4) | 0.02 | 0.006 | 0.03 |
|  | Placebo | 383.4 (324.1 - 442.7) | 298.1 (210.4 - 385.7) | 300.3 (225.0 - 375.7)^d^ |  |  |  |
| **Trabecular (4% site)**  vBMD (mg/cm^3^) | Denosumab | 257.2 (202.5 – 312.0) | 245.7 (190.9 – 300.5) | 254.9 (204.5 – 305.2) | NS | 0.011 | 0.076 |
|  | Placebo | 257.2 (182.6 - 331.9) | 229.3 (161.3 - 297.2) | 222.6 (155.6 - 289.5)^c^ |  |  |  |
| BMC (mg) | Denosumab | 146.5 (114.7 - 178.3) | 134.4 (105.7 - 163.1) | 146.4 (120.5 – 172.4) | NS | 0.02 | NS |
|  | Placebo | 135.9 (104.2 - 167.8) | 111.2 (82.5 - 139.9) | 110.2 (84.2 - 136.1) |  |  |  |
| **Total (38% site)**  vBMD (mg/cm^3^) | Denosumab | 911.5 (855.1 - 967.9) | 918.0 (862.2 - 973.9) | 915.0 (856.5 - 973.4) | 0.047 | 0.077 | NS |
|  | Placebo | 840.5 (784.1 – 896.9) | 839.1 (783.3 - 895.0) | 828.1 (769.7 - 886.6) |  |  |  |
| BMC (mg) | Denosumab | 481.6 (451.2 - 512.0) | 487.5 (452.1 - 522.9) | 490.1 (455.8 - 524.5) | 0.009 | 0.006 | NS |
|  | Placebo | 432.5 (397.8 - 467.2) | 421.4 (384.7 – 458.2) | 415.6 (378.8 – 452.4) |  |  |  |
| **Cortical (38% site)**  vBMD (mg/cm^3^) | Denosumab | 1162.0 (1117.9 – 1205.9) | 1166.6 (1132.3 - 1201.0) | 1160.8 (1125.6 – 1195.9) | 0.038 | 0.01 | NS |
|  | Placebo | 1121.8 (1077.7 - 1165.8) | 1103.1 (1068.7 – 1137.4) | 1098.9 (1063.8 - 1134.1) |  |  |  |
| BMC (mg) | Denosumab | 447.1 (413.1 - 481.1) | 454.5 (420.4 – 488.5) | 455.1 (419.4 - 490.8) | 0.008 | 0.01 | NS |
|  | Placebo | 383.8 (349.9 - 417.8) | 392.4 (358.4 - 426.5) | 385.1 (349.4 - 420.8) |  |  |  |
| **Geometry and Strength**  Area (mm) | Denosumab | 397.1 (338.6 – 455.6) | 400.3 (347.4 - 453.2) | 401.5 (354.8 – 448.2) | NS | NS | NS |
|  | Placebo | 329.6 (271.1 - 388.1) | 346.1 (293.3 – 399.0) | 342.4 (295.6 – 389.1) |  |  |  |
| CoTh (mm) | Denosumab | 4.0 (3.7 - 4.3) | 4.0 (3.7 - 4.4) | 4.0 (3.7 - 4.3) | NS | NS | NS |
|  | Placebo | 3.9 (3.6 - 4.2) | 3.9 (3.5 - 4.3) | 3.8 (3.5 - 4.1) |  |  |  |
| PC (mm) | Denosumab | 82.3 (76.3 - 88.4) | 82.6 (76.7 – 88.6) | 82.9 (77.8 – 88.1) | NS | NS | NS |
|  | Placebo | 78.4 (72.4 - 84.5) | 78.5 (72.5 - 84.5) | 78.6 (73.4 - 83.7) |  |  |  |
| EC (mm) | Denosumab | 42.7 (37.9 - 47.5) | 42.7 (37.8 - 47.7) | 43.0 (38.3 - 47.7) | NS | NS | NS |
|  | Placebo | 44.8 (40.0 - 49.6) | 42.5 (37.5 - 47.5) | 43.2 (38.5 - 47.9) |  |  |  |
| PMI (mm^4^) | Denosumab | 45,251 (39,001 – 51,499) | 46,169 (39,516 – 52,822) | 49,717 (42,665 – 56,770) | 0.04 | 0.03 | NS |
|  | Placebo | 37,073 (30,824 – 43,322) | 39,519 (32,867 - 46,172) | 38,363 (31,310 - 45,415) |  |  |  |
| SSI (mm^3^) | Denosumab | 2,370 (2,169 - 2,571) | 2,408 (2,261 - 2,555) | 2,472 (2,325 - 2,619) | 0.001 | 0.008 | NS |
|  | Placebo | 1,993 (1,791 - 2,194) | 2,058 (1,911 - 2,205) | 2,023 (1,877 - 2,170) |  |  |  |
| Values are expressed as group mean ± 95% CI and estimated marginal means where applicable. Abbreviations: SD = standard deviation; mg = milligrams; g = grams; cm = centimeters; SCI = spinal cord injury; pQCT = peripheral quantitative computed tomography; BMC = bone mineral content; vBMD = volumetric bone mineral density; CoTh = cortical thickness; PC = periosteal circumference; EC = endosteal circumference; PMI = polar moment of inertia (resistivity in torsion); SSI = stress-strain index (resistivity in bending). All BMC, vBMD, and strength index models from the 38% site were adjusted for baseline geometry variables. Significant main effects for group, visit, and interaction are indicated by p-value. Significant decrease from baseline in the placebo group: ^a^p=0.09, ^b^p=0.06, ^c^p=0.05, ^d^p=0.009. | | | | | | | |
